# Routine Oxygen Therapy Does Not Improve Health-Related Quality of Life in Patients With Acute Myocardial Infarction—Insights From the Randomized DETO2X-AMI Trial

**DOI:** 10.3389/fcvm.2021.638829

**Published:** 2021-03-15

**Authors:** Robin Hofmann, Tamrat Befekadu Abebe, Johan Herlitz, Stefan K. James, David Erlinge, Troels Yndigegn, Joakim Alfredsson, Thomas Kellerth, Annica Ravn-Fischer, Sebastian Völz, Jörg Lauermann, Tomas Jernberg, Bertil Lindahl, Sophie Langenskiöld

**Affiliations:** ^1^Division of Cardiology, Department of Clinical Science and Education, Karolinska Institutet, Södersjukhuset, Stockholm, Sweden; ^2^Department of Medical Sciences, Uppsala University, Uppsala, Sweden; ^3^Department of Health Sciences, University of Borås, Borås, Sweden; ^4^Uppsala Clinical Research Center, Uppsala University, Uppsala, Sweden; ^5^Department of Clinical Sciences, Cardiology, Lund University, Lund, Sweden; ^6^Department of Health, Medicine and Caring Sciences, Linköping University, Linköping, Sweden; ^7^Department of Cardiology, Linköping University Hospital, Linköping, Sweden; ^8^Department of Cardiology, Faculty of Medicine and Health, Örebro University, Örebro, Sweden; ^9^Department of Molecular and Clinical Medicine, Sahlgrenska University Hospital, Gothenburg, Sweden; ^10^Department of Cardiology, University of Gothenburg, Gothenburg, Sweden; ^11^Department of Cardiology, Ryhov Hospital, Jönköping, Sweden; ^12^Department of Health, Medicine and Caring, Linköping University, Linköping, Sweden; ^13^Department of Clinical Sciences, Cardiology, Karolinska Institutet, Danderyd Hospital, Stockholm, Sweden

**Keywords:** myocardial infarction, oxygen therapy, Registry-based randomized clinical trial, secondary prevention, health-realted quality of life, patient reported clinical outcomes

## Abstract

**Background:** After decades of ubiquitous oxygen therapy in all patients with acute myocardial infarction (MI), recent guidelines are more restrictive based on lack of efficacy in contemporary trials evaluating hard clinical outcomes in patients without hypoxemia at baseline. However, no evidence regarding treatment effects on health-related quality of life (HRQoL) exists. In this study, we investigated the impact of routine oxygen supplementation on HRQoL 6–8 weeks after hospitalization with acute MI. Secondary objectives included analyses of MI subtypes, further adjustment for infarct size, and oxygen saturation at baseline and 1-year follow-up.

**Methods:** In the DETermination of the role of Oxygen in suspected Acute Myocardial Infarction (DETO2X-AMI) trial, 6,629 normoxemic patients with suspected MI were randomized to oxygen at 6 L/min for 6–12 h or ambient air. In this prespecified analysis, patients younger than 75 years of age with confirmed MI who had available HRQoL data by European Quality of Life Five Dimensions questionnaire (EQ-5D) in the national registry were included. Primary endpoint was the EQ-5D index assessed by multivariate linear regression at 6–10 weeks after MI occurrence.

**Results:** A total of 3,086 patients (median age 64, 22% female) were eligible, 1,518 allocated to oxygen and 1,568 to ambient air. We found no statistically significant effect of oxygen therapy on EQ-5D index (−0.01; 95% CI: −0.03–0.01; *p* = 0.23) or EQ-VAS score (−0.57; 95% CI: −1.88–0.75; *p* = 0.40) compared to ambient air after 6–10 weeks. Furthermore, no significant difference was observed between the treatment groups in EQ-5D dimensions. Results remained consistent across MI subtypes and at 1-year follow-up, including further adjustment for infarct size or oxygen saturation at baseline.

**Conclusions:** Routine oxygen therapy provided to normoxemic patients with acute MI did not improve HRQoL up to 1 year after MI occurrence.

**Clinical Trial Registration:** ClinicalTrials.gov number, NCT01787110.

## Introduction

For decades, oxygen therapy has been used liberally in patients with acute myocardial infarction (MI) across the world (1). The rationale behind this widespread use was to optimize oxygen delivery to the ischemic heart muscle with the goal of reducing infarct size as well as potential complications such as heart failure and malignant arrhythmias (2). Additionally, oxygen was believed to reduce pain, anxiety, and nausea (3). Overall, health care professionals provided supplemental oxygen with the strong conviction to improve patients' outcomes and quality of life after MI (4). In contrast, experimental (2, 5–7) and clinical (8) evidence showed that high arterial oxygen content may lead to negative cardiovascular effects and potentially detrimental outcomes including increased morbidity and mortality (9).

To elucidate this controversy, the Determination of the role of Oxygen in suspected Acute Myocardial Infarction (DETO2X-AMI) trial compared routine oxygen therapy with ambient air in 6,629 normoxemic patients with suspected MI. We did not find any clinically significant effect of supplemental oxygen on survival or cardiovascular outcomes in the short or long-term perspective (10, 11), nor could we demonstrate any analgesic or sedative effect (12). Results were robust across relevant subgroups with suspected (10, 13) and confirmed MI (14–17).

Experts have been requesting evaluation of the impact of oxygen therapy on health-related quality of life (HRQoL) post-MI (18) based on the established association between cognitive deficits and functional disability after MI (19), arguing that this phenomenon could be caused by cerebral hypoxemia, which can occur despite peripheral normoxemia, in particular in patients with hypertension or who are elderly (20).

To the best of our knowledge, no data exist on the impact of oxygen therapy on HRQoL in normoxemic patients after MI. In this prespecified subgroup analysis from the DETO2X-AMI trial involving 3,086 patients with confirmed MI, we therefore investigated the impact of routine oxygen supplementation on HRQoL 6–10 weeks after hospitalization. Secondary objectives included analyses of MI subtypes and 1-year follow-up.

## Methods

### Study Design

The DETO2X-AMI study was a multicenter, parallel-group, open-label, registry-based, randomized controlled trial (RRCT) (21) comparing oxygen therapy with ambient air in normoxemic patients with suspected MI (22). The national comprehensive Swedish Web System for Enhancement and Development of Evidence-Based Care in Heart Disease Evaluated According to Recommended Therapies (SWEDHEART) (23) was used for data collection, and follow-up.

Regional Ethical Review Board in Gothenburg (DNR 287-12) and the medical products agency of Sweden (EudraCT 2013-002882-20) approved the study. Trial sponsor was Karolinska Institutet, Stockholm, Sweden. The design, methods, and primary results have been published previously (10, 11, 22).

The sources of funding had no role in design and conduct of the study; collection, management, analysis, and interpretation of the data; preparation, review, or approval of the manuscript; and decision to submit the manuscript for publication.

### Patient Population

At first, medical contact with the ambulance service, emergency department, coronary care unit, or catheterization laboratory of participating hospitals' patients were evaluated for enrollment. Inclusion criteria included patients who were ≥30 years of age with symptoms suggestive of acute MI (defined as chest pain or shortness of breath) for <6 h with an oxygen saturation of ≥90% on pulse oximetry, and either electrocardiographic changes indicating ischemia (24) or elevated cardiac troponin on admission. To allow complete follow-up through the Swedish National Population Registry (25), only residents of Sweden with a unique personal identification number were enrolled. Patients on continuous oxygen therapy or with cardiac arrest prior to evaluation were excluded.

The overall study population included patients with suspected MI (*N* = 6,629). One fourth of these patients received other discharge diagnosis than acute MI (10). The present study describes a prespecified subgroup analysis in patients who were followed up in clinical routine over 1 year in the Secondary Prevention after Heart Intensive Care Admission (SEPHIA) part of SWEDEHEART where only patients with confirmed MI below the age of 75 are subject for follow-up, comprising 3,086 individuals in our study population. Based on the fact that background characteristics, the in-hospital course, secondary prevention, as well as short- and long-term prognosis differ between MI subtypes (26–28), and consequently may impact on HRQoL post-MI, we further stratified the study population by MI subtype into ST-elevation myocardial infarction (STEMI) and Non-STEMI (NSTEMI).

### Procedure and Data Collection

Following oral informed consent, eligible patients were randomly assigned in a 1:1 ratio to either oxygen therapy at 6 L/min for 6–12 h given by open face mask or ambient air. Randomization was performed online linked to the SWEDHEART database. The randomized treatment was started directly after randomization. Oral consent was confirmed by signature within 24 h. All patients were treated according to standard of care. Oxygen saturation was documented at the beginning and the end of the randomized treatment period.

Data on patients' health-related quality of life (HRQoL) were retrieved from SEPHIA. The registry captures HRQoL using the European Quality of Life Five Dimensions questionnaire (EQ-5D) for patients younger than 75 with confirmed MI 6–10 weeks (visit 1), and 12–14 months (visit 2) after the MI occurrence. The EQ-5D has been used in several studies on cardiovascular disease and is considered a valid instrument in this population (29). It is a generic instrument that measures patients' HRQoL from five dimensions (mobility, self-care, usual activities, pain/discomfort, and anxiety/depression). The patients self-rate their current health state using a three-item ordinal response scale (no/moderate/severe problem) (30). Their rating results in any of the 243 (3^5^) possible health states, which can be translated to a quality-of-life weight (EQ-5D index) using any of the available tariffs. For the present study, we used the UK tariff to generate the EQ-5D index (31). The UK tariff ranges from the worst possible health state −0.59 to the best health state 1.00 (31).

The EQ-5D also includes a visual analog scale (EQ-VAS), a vertically arranged VAS with the scale ranging from 0 (the worst health you can imagine) to 100 (the best health you can imagine). EQ-VAS captures broader underlying constructs of health and provides a summarized health status that is closer to the patients' perspective (32).

No baseline HRQoL data were available since data in SEPHIA are only registered at the aforementioned follow-up visits.

### Endpoints and Follow-Up

The primary outcome was EQ-5D index at 6–8 weeks (visit 1) in MI patients stratified by randomized treatment (oxygen/ambient air). We only proceeded to analyze the second visit after 12–14 months if we observed a potentially significant trend at the first visit.

Secondary outcome included EQ-VAS score and EQ-5D dimensions.

Outcomes were analyzed in the total population and stratified according to MI subtype (STEMI/NSTEMI).

As sensitivity analysis, to explore the robustness of our findings, we adjusted for infarct size assessed by the highest measured level of high-sensitivity cardiac troponin (hs-cTn) T and oxygen saturation at baseline, factors known to impact on prognosis (17) and, thus, possibly also the patients' HRQoL.

### Covariates

This study comprises a predefined subgroup analysis of the randomized DETO2X-AMI study population. Consequently, the randomization may not have controlled for all differences across the groups, which are compared in this study. Multivariate adjustment was used to control for known or suspected confounders, including sociodemographic variables (age, sex), smoking status, comorbidities (hypertension, diabetes), history of MI, and/or revascularization [coronary artery bypass graft (CABG) or percutaneous coronary intervention (PCI)]; vital signs at presentation (systolic blood pressure, heart rate), medications on admission (aspirin, P2Y12 receptor inhibitor, beta-blocker, statin, angiotensin converting enzyme inhibitor or angiotensin II blocker, calcium channel blocker, and diuretics), type of MI (STEMI, NSTEMI), ambulance transportation, and body mass index (BMI) were included in the model.

### Statistical Analysis

Numerical variables are presented as arithmetic mean (±SD) and categorical variables are presented as count (%) unless otherwise specified. Results were analyzed based on the intention-to-treat principle. Mean scores for EQ-5D index and EQ-VAS were reported and further explored by applying multivariate linear regression adjusting for important covariates mentioned above. Distribution of EQ-5D index and EQ-VAS score between the treatment groups was reported using an empirical cumulative distribution function (ECDF) plot. The categorical outcome EQ-5D dimensions was evaluated by Fisher's exact test.

A *p* < 0.05 was considered statistically significant.

Data pre-processing and analysis were performed in R version 3.6.2.

## Results

### Patient Population

**Figure 1:**
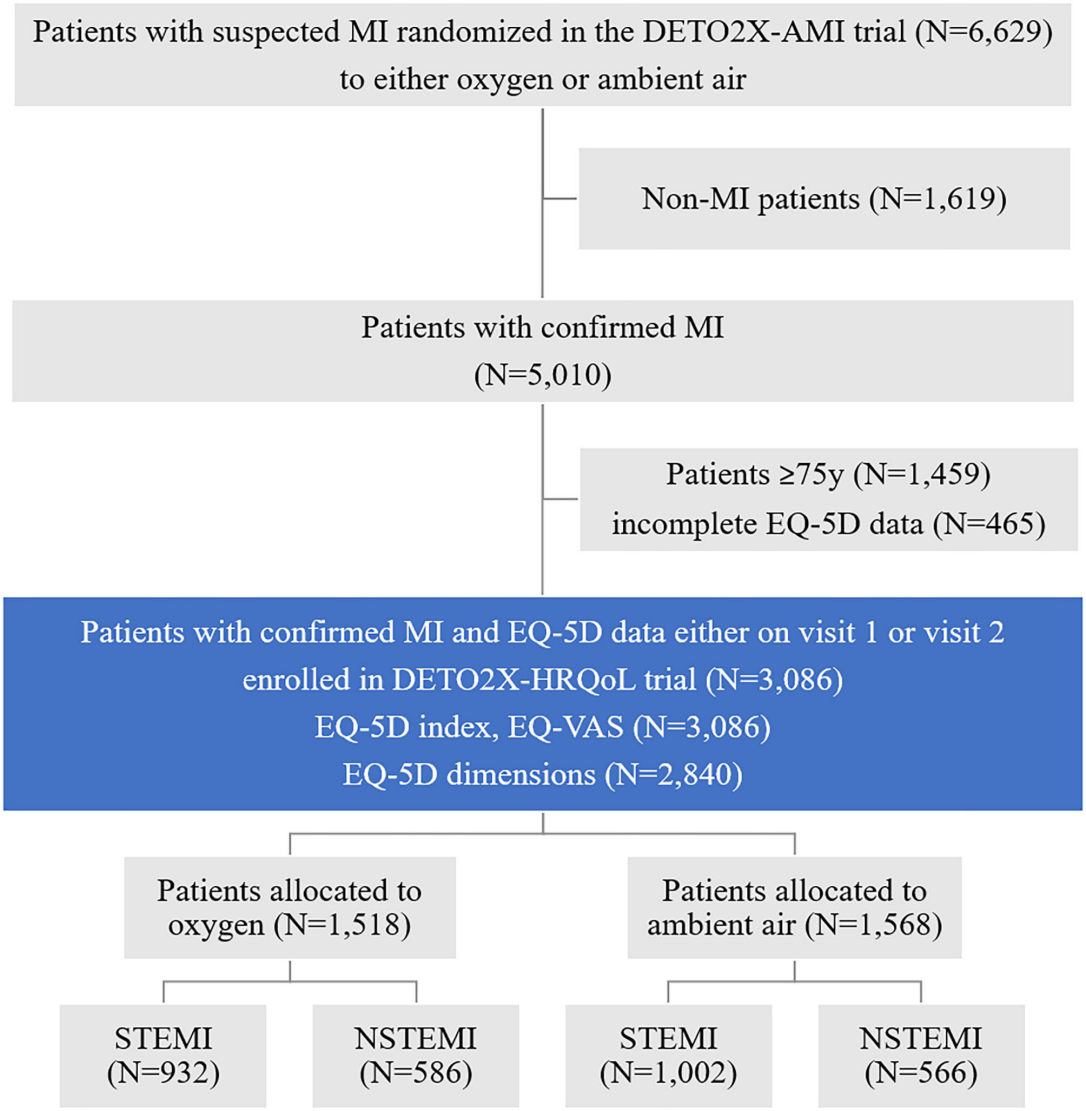
Enrollment, randomization, and analysis (study flow chart). Eligible patients with suspected myocardial infarction were evaluated for inclusion in the main study, randomly assigned to a study group (oxygen vs. ambient air), and analyzed during the study period. In the present prespecified subgroup analysis, only patients with confirmed MI below the age of 75 who are subject for follow-up in the national registry were included. DETO2X-AMI, Determination of the Role of Oxygen in Acute Myocardial Infarction; MI, myocardial infarction; EQ-5D, European Quality of Life Five Dimensions questionnaire; DETO2X-HRQoL, DETO2X health-related quality of life subgroup analysis; STEMI, ST-elevation myocardial infarction; NSTEMI, Non-ST-elevation myocardial infarction.

Between April 13, 2013, and December 30, 2015, a total of 6,629 patients with suspected MI were enrolled in the main study, of which 5,010 (75.5%) were discharged with confirmed MI. Of these, 3,086 patients were <75 years and had HRQoL data either on the first or second SEPHIA follow-up visit recorded. A total of 1,518 were randomized to oxygen, and 1,568 were randomized to ambient air (flow chart, Figure 1). Baseline characteristics and clinical presentation of the study participants were well-balanced except for the mean systolic blood pressure, which was significantly higher in the oxygen group (*p* = 0.01). The final diagnosis was STEMI in 932 (61.4%) and 1,002 (63.9%) patients in the oxygen and ambient-air group, respectively. All remaining patients were diagnosed with NSTEMI (Table 1).

**Table 1:** Baseline characteristics, clinical presentation, and final diagnosis.

**Characteristics**	**Oxygen**	**Ambient air**
	***N* = 1,518**	***N* = 1,568**
Age, median (IQR), years	63 (56–69)	64 (56–69)
Male sex, *n* (%)	1,167 (76.9)	1,238 (79.0)
Body mass index	27.7 ± 4.45	27.5 ± 4.34
Current smoker, *n* (%)	451 (29.7)	472 (30.1)
Hypertension, *n* (%)	624 (41.2)	615 (39.2)
Diabetes mellitus, *n* (%)	239 (15.7)	268 (17.1)
**Previous cardiovascular disease**, ***n*** **(%)**		
Myocardial infarction	179 (11.8)	198 (12.6)
PCI	161 (10.6)	168 (10.7)
CABG	44 (2.9)	52 (3.32)
**Cause of admission**, ***n*** **(%)**		
Chest pain	1,470 (99.2)	1,522 (98.9)
Dyspnea	11 (0.74)	17 (1.1)
Cardiac arrest	1 (0.07)	0
**Medication at admission**, ***n*** **(%)**		
Aspirin	267 (17.7)	310 (19.8)
P2Y_12_ receptor inhibitor	31 (2.04)	29 (1.85)
Beta-blocker	303 (20.1)	353 (22.6)
Statin	302 (20.0)	326 (20.8)
ACE inhibitor or angiotensin-II blocker	459 (30.5)	462 (29.6)
Calcium blocker	235 (15.6)	235 (15.1)
Diuretic	128 (8.5)	128 (8.21)
Median time from symptom onset to randomization (IQR), min	210 (120–390)	217 (118–413)
Ambulance transportation, *n* (%)	1,029 (67.8)	1,044 (66.6)
**Vital signs at presentation**		
Systolic blood pressure, mm Hg^*^	151 ± 27.60	149 ± 27.90
Heart rate, beats/min	77.1 ± 17.70	76.5 ± 18.00
Median oxygen saturation (IQR), %	98 (96–99)	97 (96–98)
Final diagnosis, *n* (%)		
STEMI	932 (61.4)	1,002 (63.9)

* *p < 0.05*.

*ACE, angiotensin-converting enzyme; CABG, coronary artery bypass graft; IQR, interquartile range; PCI, percutaneous coronary intervention; STEMI, ST-segment elevation myocardial infarction*.

### Procedural Data

Data on medication, procedures, and complications during the hospitalization period are presented in Table 2. As expected, the median oxygen saturation at the end of treatment period was significantly different between patients who were assigned to oxygen (99%) or ambient air (97%) (*p* < 0.001).

**Table 2:** Data on procedures, medication, and complications during hospitalization.

	**Oxygen**	**Ambient air**
	***N* = 1,518**	***N* = 1,568**
Duration of oxygen therapy, median (IQR), h	11.8 (6.1–12.0)	
Median oxygen saturation at end of treatment period (IQR), %^*^	99 (97–100)	97 (96–98)
Hypoxemia, (%)^*^	30 (2.0)	111 (7.1)
**Procedures**, ***n*** **(%)**		
Coronary angiography	1,502 (98.9)	1,557 (99.3)
PCI	1,360 (90.0)	1,421 (90.6)
CABG	38 (2.5)	57 (3.6)
Median duration of hospital stay (range), days	3 (0–68)	3 (0–71)
**Medication**, ***n*** **(%) Intravenous medications**		
Intravenous diuretic	94 (6.2)	109 (7.0)
Intravenous inotrope	22 (1.5)	28 (1.8)
Intravenous nitroglycerin	123 (8.1)	112 (7.1)
**Discharge medications**		
Aspirin	1,460 (96.2)	1,519 (96.9)
P2Y_12_ receptor inhibitor	1,451 (95.6)	1,483 (94.6)
Beta-blocker^*^	1,377 (90.7)	1,454 (92.7)
Statin	1,490 (98.2)	1,534 (97.8)
ACE inhibitor or angiotensin II blocker	1,338 (88.1)	1,356 (86.5)
Calcium blocker	179 (11.8)	180 (11.5)
Diuretic	163 (10.7)	173 (11.0)
**Complication**, ***n*** **(%)**		
Reinfarction	9 (0.6)	5 (0.32)
New-onset atrial fibrillation	36 (2.4)	37 (2.36)
Atrioventricular block, 2nd, or 3rd degree	30 (2.0)	23 (1.47)
Cardiogenic shock	12 (0.8)	13 (0.8)
Cardiac arrest	32 (2.1)	27 (1.7)

* *p < 0.05*.

*ACE, angiotensin-converting enzyme; CABG, coronary artery bypass graft; IQR, interquartile range; PCI, percutaneous coronary intervention; STEMI, ST-segment elevation myocardial infarction*.

Hypoxemia necessitating administration of oxygen outside the protocol developed in 111 (7.0%) patients in the ambient-air group and 30 (2.0%) patients in the oxygen group (*p* < 0.001). The frequency of in-hospital procedures, medication, and complications was evenly distributed between the randomized groups except for the use of beta-blockers at discharge, which was higher in the ambient-air group compared to the oxygen group (*p* = 0.03).

### HRQoL Outcomes

#### Main Analysis, Visit 1 (6–10 Weeks)

**Figure 2:**
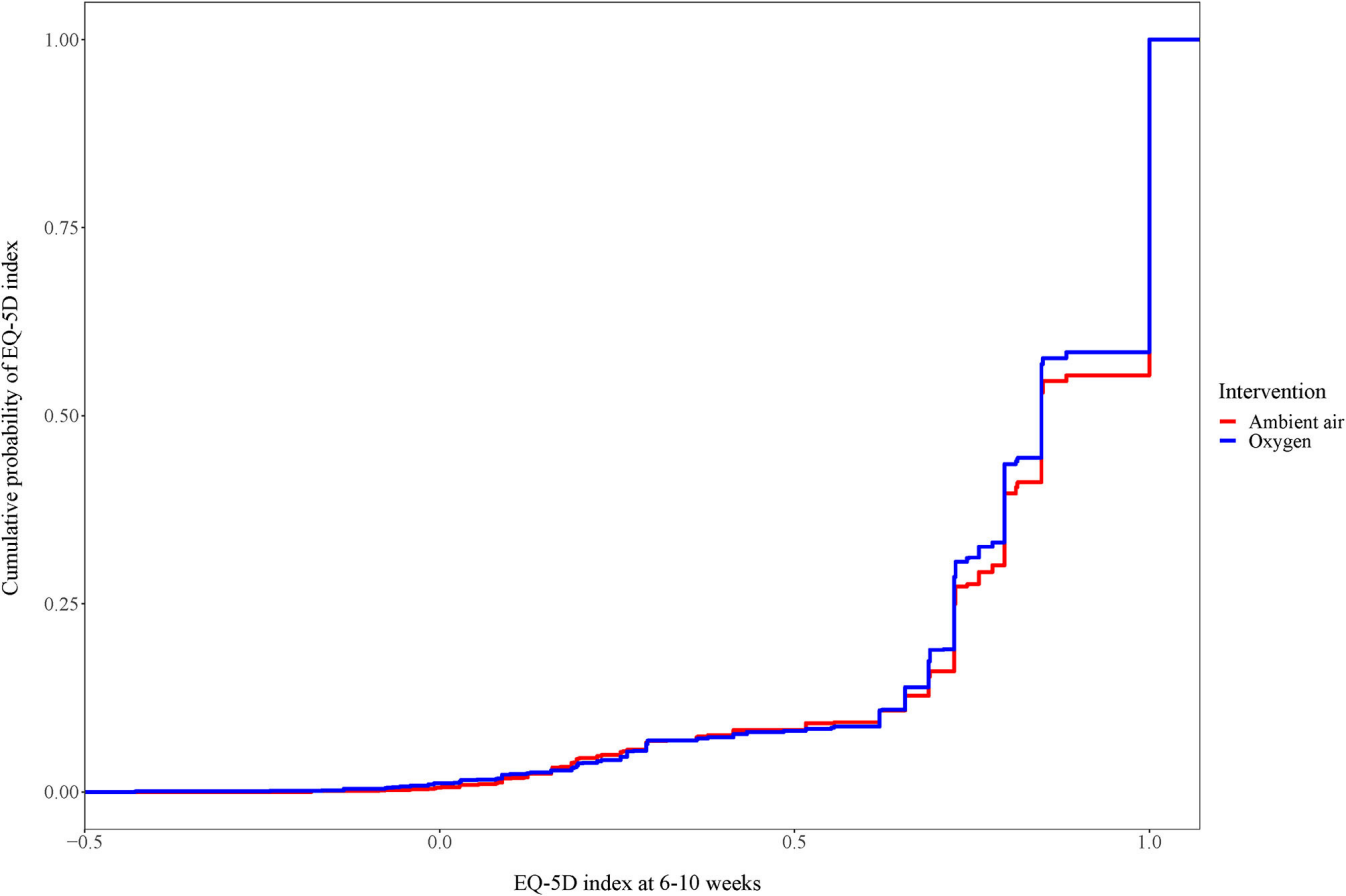
Cumulative distribution of EQ-5D index for patients with confirmed myocardial infarction at weeks 6–10. Cumulative distribution of EQ-5D index. For each EQ-5D index value displayed, the graph shows the proportion of patients below that cut point.

The mean EQ-5D index was 0.82 (SD ±0.23) in the oxygen group compared to 0.83 (SD ±0.23) in the ambient-air group. In the multivariate linear regression analysis, oxygen treatment had no impact on the EQ-5D index (−0.01; 95% CI: −0.03–0.01; *p* = 0.23) (Table 3). Displayed as the cumulative probability per randomized treatment, the curves were almost superimposed on each other (Figure 2). Due to the neutral results, we did not analyze results from visit 2.

**Table 3:** Multivariate linear regression model of EQ-5D index and EQ-VAS score at 6–10 weeks for patients with confirmed MI.

	**EQ-5D index**			**EQ-VAS score**		
**Coefficient**	**Estimates**	**95% CI**	***p*-value**	**Estimates**	**95% CI**	***p*-value**
Intercept	0.82	0.71–0.92	<0.001	69.09	60.63 to 77.55	<0.001
**Intervention (Oxygen)**	**−0.01**	**−0.03 to 0.01**	**0.23**	**−0.57**	**−1.89 to 0.75**	**0.40**
Age (years)	0.00	0.00 to 0.00	<0.001	0.23	0.15 to 0.32	<0.001
Gender (female)	−0.06	−0.08 to −0.04	<0.001	−2.37	−3.99 to −0.75	<0.01
Body Mass Index (kg/m^2^)	−0.00	−0.00 to −0.00	<0.01	−0.26	−0.42 to −0.10	<0.01
Smoking (yes)	−0.05	−0.07 to −0.03	<0.001	−3.26	−4.76 to −1.76	<0.001
Hypertension (yes)	−0.02	−0.04 to 0.00	0.11	−1.74	−3.66 to 0.19	0.08
Diabetes (yes)	−0.03	−0.06 to −0.01	0.01	−2.09	−4.07 to −0.11	0.04
Previous MI (yes)	−0.06	−0.10 to −0.01	0.02	−6.00	−9.63 to −2.38	0.001
Previous PCI (yes)	0.04	−0.00 to 0.09	0.07	2.62	−1.16 to 6.41	0.18
Prior CABG (yes)	0.05	−0.00 to 0.11	0.05	0.47	−3.76 to 4.70	0.83
Aspirin (yes)	−0.03	−0.06 to 0.00	0.09	1.70	−0.69 to 4.08	0.16
Beta-blockers (yes)	−0.01	−0.04 to 0.01	0.37	−1.09	−3.15 to 0.97	0.30
Statins (yes)	−0.01	−0.04 to 0.02	0.40	−1.83	−3.95 to 0.29	0.09
ACE/ARB (yes)	0.01	−0.01 to 0.04	0.35	2.41	0.47 to 4.34	0.02
CCB (yes)	−0.00	−0.03 to 0.03	0.99	−0.25	−2.31 to 1.82	0.82
Diuretic (yes)	−0.06	−0.09 to −0.03	<0.001	−4.57	−7.17 to −1.96	0.001
Other antiplatelets (yes)	−0.03	−0.09 to 0.04	0.45	−4.68	−9.86 to 0.50	0.08
Ambulance service (yes)	0.00	−0.02 to 0.02	0.95	−0.98	−2.45 to 0.48	0.19
Systolic blood pressure (mmHg)	0.00	0.00 to 0.00	0.001	0.04	0.02 to 0.07	0.001
Heart rate (beats/min)	−0.00	−0.00 to −0.00	0.04	−0.05	−0.09 to −0.01	0.01
MI subtype (NSTEMI)	−0.01	−0.03 to 0.01	0.41	0.57	−0.88 to 2.02	0.44
Observations	2,725	2,717				
*R*^2^/*R*^2^ adjusted	0.07/0.06	0.06/0.06				

*ACE, angiotensin-converting enzyme; ARB, angiotensin receptor blocker; CABG, coronary artery bypass graft; CCB, calcium channel blockers; MI, myocardial infarction; PCI, percutaneous coronary intervention; STEMI, ST-segment elevation myocardial infarction. This was intended to highlight the main effect of the intervention (oxygen)*.

**Figure 3:**
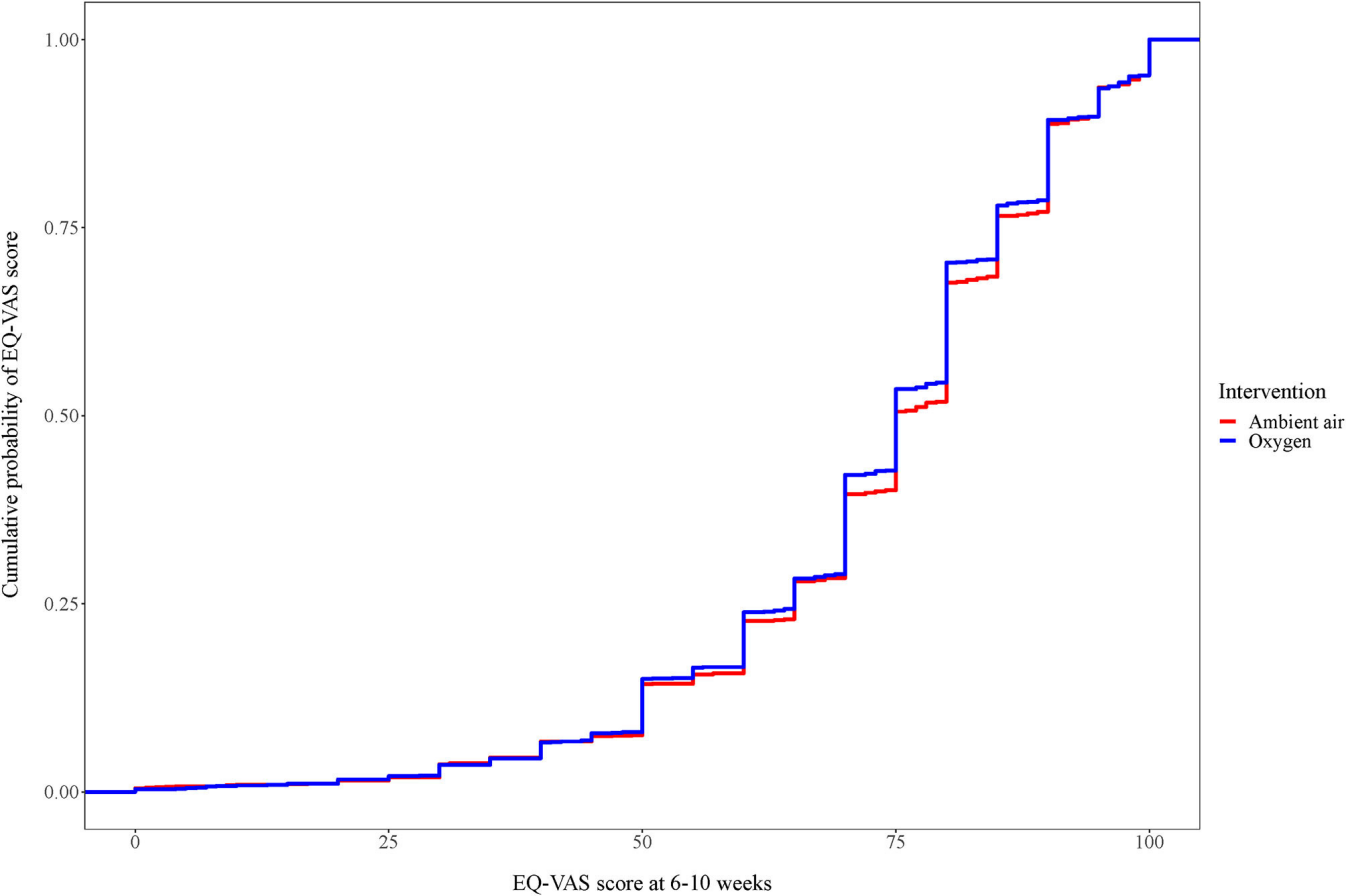
Cumulative distribution of EQ-VAS for patients with confirmed myocardial infarction at weeks 6–10. Cumulative distribution of EQ-VAS. For each EQ-5D index value displayed, the graph shows the proportion of patients below that cut point.

The mean EQ-VAS score was 72.90 (SD ±18.00) in the oxygen group compared to 73.50 (SD ±18.10) in the ambient-air group. In the multivariate linear regression analysis, oxygen treatment had no impact on the EQ-VAS score (0.57; 95% CI: −1.88–0.75; *p* = 0.40) (Table 3, Figure 3).

**Figure 4:**
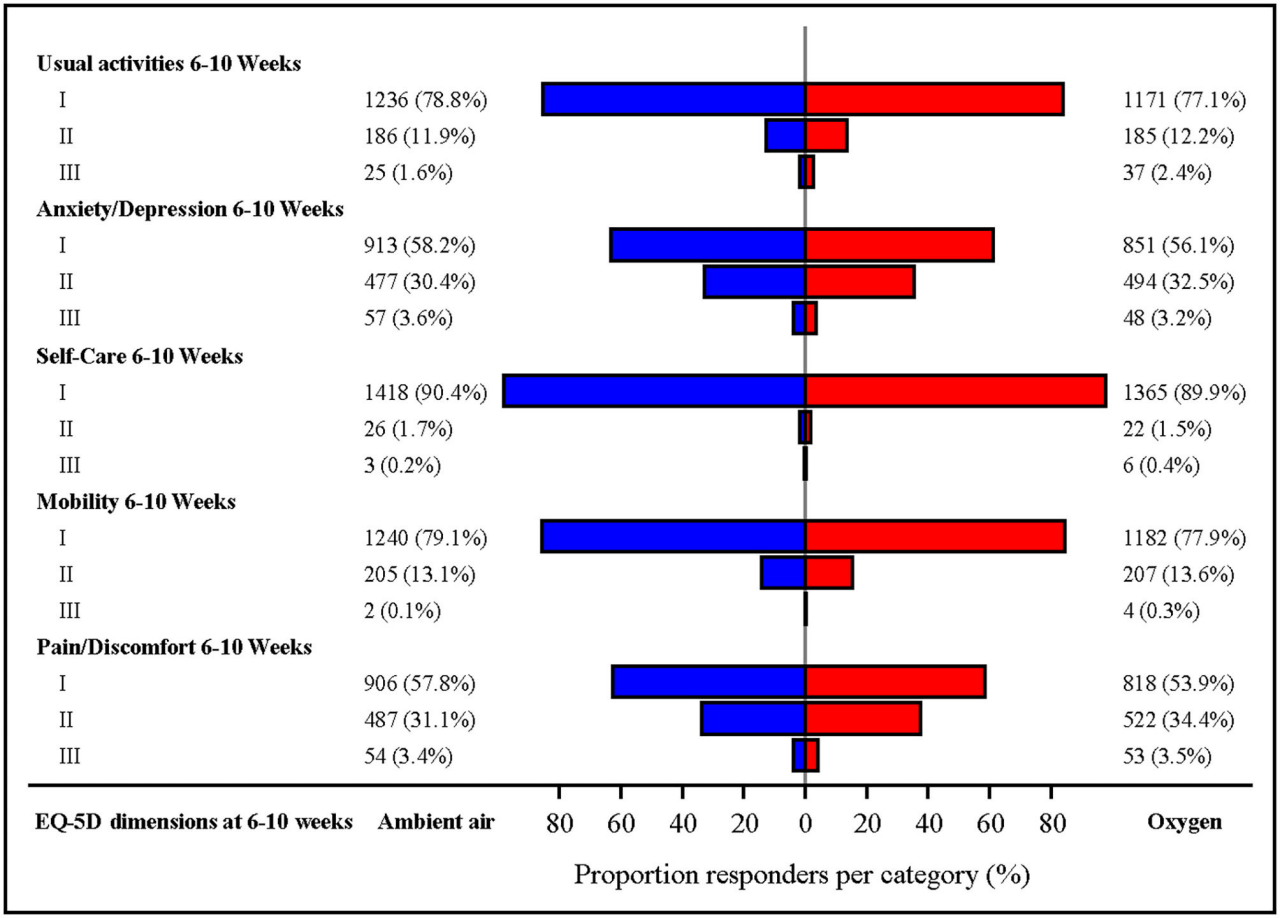
EQ-5D dimensions on visit 1. The EQ-5D is a generic instrument that measures patients' health-related quality of live five dimensions (mobility, self-care, usual activities, pain/discomfort, and anxiety/depression). The patients' self-rate their current health state using a three-item ordinal response scale (no/moderate/severe problem). *p*-values for comparison of oxygen vs. ambient air were non-significant for all dimensions. Missing observations: 8.2% in the oxygen group and 7.7% in the ambient-air group.

The EQ-5D dimensions were similar between the randomized groups (Figure 4).

Due to the overall neutral results, we did not analyze results from visit 2 (12–14 months).

### Subgroup Analyses

#### STEMI Subgroup

In patients with STEMI at visit 1 (6–10 weeks), the mean EQ-5D index was 0.81 (±0.23) in the oxygen group compared to 0.83 (±0.22) in the ambient-air group. In the multivariate linear regression analysis, we observed a trend toward reduced EQ-5D index in the patient allocated to oxygen treatment (0.02; 95% CI: −0.04–0.00; *p* = 0.06) compared to ambient air (Supplementary Table I). The mean EQ-VAS score was 71.97 (SD ±18.10) in the oxygen group compared to 73.47 (SD ±18.20) in the ambient-air group. In the multivariate linear regression analysis, oxygen treatment reduced the EQ-VAS score but without statistical significance (−1.33; 95% CI: −3.00–0.34; *p* = 0.12) (Supplementary Table I). The EQ-5D dimensions showed significant differences in the pain/discomfort domain with a higher score (= more pain/discomfort) in the patients allocated to oxygen (*p* = 0.04) (Supplementary Table II).

Due to the trend toward a difference in EQ-5D index at visit 1, we moved forward and analyzed results from visit 2 (12–14 months). At that time point, the mean EQ-5D index was 0.82 (SD ±0.23) in the oxygen therapy group and 0.84 (SD ±0.21) in the ambient-air group. In the multivariate linear regression analysis, receiving oxygen therapy significantly lowered the EQ-5D index (−0.02; 95% CI: −0.04–0.00); *p* = 0.03) compared to ambient air (Supplementary Table III). EQ-VAS was not analyzed at visit 2. None of the EQ-5D dimensions were statistically significant between the randomized groups (Supplementary Table IV).

#### NSTEMI Subgroup

None of the HRQoL outcomes differed statistically significant in either multivariate linear regression models (EQ-5D index or EQ-VAS score; Supplementary Table V) or EQ-5D dimensions (Supplementary Table VI).

### Sensitivity Analysis

Sensitivity analysis using multivariate linear regression also including highest measured level of hs-cTn T and oxygen saturation at baseline showed consistent results for the whole population, as well as stratified to MI subtypes (Supplementary Tables VII–IX).

## Discussion

In contrast to clinical practice over the last decades, contemporary randomized clinical trials did not show any prognostic benefit of routine oxygen therapy regarding survival or cardiovascular outcomes compared to ambient air in patients with acute MI without hypoxemia at baseline (10, 11, 33). Accordingly, in the present study, we found that HRQoL assessed by EQ-5D index, EQ-5D-VAS, and EQ-5D dimensions was similar in patients with confirmed MI regardless of allocated treatment over a follow-up of 1 year after MI occurrence. These findings remained consistent across MI subtypes and in a sensitivity analysis adjusting for MI severity by infarct size and baseline oxygen saturation.

Based on the results of recent trials evaluating the role of supplemental oxygen in normoxemic patients with MI, guidelines throughout the world have been changed to a more restrictive use of oxygen therapy, now exclusively recommended in patients with hypoxemia at baseline (defined as oxygen saturation <90%) (26, 27, 34). Some clinicians remain skeptical, however, arguing that limitations to the underlying evidence may warrant new trials (18, 35). To our knowledge, the current analysis is the first randomized clinical trial assessing the effects of oxygen therapy on HRQoL in the setting of acute MI. Thus, it is challenging to put our findings into perspective, leaving us to a comparison in more general terms. A recent study from SWEDEHEART on 27,267 consecutive patients with a first-time MI assessing emotional stress using the EQ-5D domain from the SEPHIA follow-up reported previous depression/anxiety, female gender, younger age, smoking, and readmission due to cardiovascular events as factors strongly associated with emotional distress post-MI (36). Unfortunately, no data on EQ-5D index, EQ-5D VAS, or EQ-5D dimensions were reported, but we utilized the identified risk factors in the adjusted analyses presented here. When comparing the EQ-5D index of the current study with the general population of Sweden, results were similar (37). In prospective cohort studies from Switzerland and Malaysia assessing HRQoL in patients with acute coronary syndrome (including unstable angina who were excluded in our trial), the mean EQ-5D index and EQ-VAS score were also similar to our results (38, 39). Noteworthy, the statistical models used in the Swiss trial and others (40) aligned well with ours concerning effects of covariates on HRQoL, which adds validity to our results and the comparison across studies. Despite the fact that previous trials neither adjusted for oxygen saturation at baseline nor evaluated treatment effects of supplemental oxygen, their results add circumstantial evidence in agreement with our results that routine oxygen therapy does not significantly affect HRQoL in patients after MI.

In a subgroup analysis, oxygen-treated patients with STEMI showed a trend toward lower EQ-5D index at 6–10 weeks after MI, which reached statistical significance after 1 year. A previous study evaluating HRQoL in MI patients reported a change of 0.09 units in the EQ-5D index as clinically meaningful (41). Consequently, the difference that we observed to the disadvantage of oxygen treatment in STEMI patients was too small to have any clinical importance. STEMI patients in the oxygen group also had a slightly higher proportion of moderate and severe pain/discomfort compared to the ambient-air group at visit 1 that dissipated onto visit 2. Again, the clinical relevance is questionable. Previous studies could not find any evidence confirming an analgesic effect of supplemental oxygen in the acute setting with PCI-treated angina (42), or MI, regardless of MI subtype (12).

Of interest with regard to the present analysis is the question of the association between cognitive deficits and functional disability after MI due to undetected cerebral hypoxemia (19). Despite lack of variables directly assessing neurologic function available from the SWEDEHEART registry, the assessment of HRQoL as presented here may serve as a valid proxy. We did not find any signal indicating improved HRQoL measures by routine oxygen treatment. Our findings are in agreement with a recently reported multicenter, randomized trial from United Kingdom involving 8,003 patients with acute stroke, where routine prophylactic low-dose oxygen therapy did not affect the primary endpoint of 90-day functional and neurological outcome (43).

Strengths of our trial include using the generic, well-established EQ-5D index, which allows comparison across different diseases summarizing the patient self-reported health perception (44). The large sample size from a pragmatic randomized trial and accuracy of our findings have good potential to be generalizable to clinical practice.

Despite the above strengths, our study has some limitations. First, EQ-5D was only available in patients <75 years of age excluding a priori an important group of patients. Second, we did not have EQ-5D data at baseline, which made it impossible to adjust for HRQoL prior to MI. In particular, EQ-VAS score was considered exploratory as no baseline measurement was available to control for different biases common with the VAS scale [context bias (45) and end of state aversion (46)]. Third, lack of disease-specific patient-reported outcomes did not allow us to capture small but potentially important disease-related changes in HRQoL.

## Conclusion

Routine oxygen therapy provided to normoxemic patients with acute MI did not improve HRQoL up to 1 year after MI occurrence, regardless of MI subtype, which corroborates current international guidelines (26, 27, 34). Future studies should also pay attention to the cost of providing oxygen unnecessarily and how much resources and time could potentially be saved by limiting oxygen therapy to patients with hypoxemia.

## Data Availability Statement

The datasets presented in this article are not readily available because the data set is derived from the SWEDEHEART registry which has separate rules for data sharing. Requests to access the datasets should be directed to www.swedeheart.se.

## Ethics Statement

The studies involving human participants were reviewed and approved by Regional Ethical Review Board in Gothenburg, Sweden (DNR 287-12). The patients/participants provided their written informed consent to participate in this study.

## Author Contributions

RH, TB, BL, and SL had full access to all of the data in the study and take responsibility for the integrity of the data and the accuracy of the data analysis. RH, TB, BL, and SL: concept and design, drafting of the manuscript, statistical analysis, administrative, technical, or material support, and supervision. RH and JH: obtained funding. All authors: acquisition, analysis, or interpretation of data, and critical revision of the manuscript for important intellectual content.

## Conflict of Interest

The authors declare that the research was conducted in the absence of any commercial or financial relationships that could be construed as a potential conflict of interest.
