# Supplementary material for: Routine Oxygen Therapy Does Not Improve Health-Related Quality of Life in Patients With Acute Myocardial Infarction—Insights From the Randomized DETO2X-AMI Trial
Source: Front Cardiovasc Med. 2021 Mar 15;8:638829. doi: 10.3389/fcvm.2021.638829 (PMC8006541; doi:10.3389/fcvm.2021.638829)
Supplement: Supplementary file 1 [file Data_Sheet_1.DOCX]

Appendix

Supplemental material to:

**Routine oxygen therapy does not improve health-related**

**quality of life in patients with acute myocardial infarction -**

**insights from the randomized DETO2X-AMI trial**

*Robin Hofmann***, M.D., Ph.D.^1^; Tamrat Befekadu Abebe***,^2^; Johan Herlitz, M.D., Ph.D.^3^; Stefan K. James, M.D., Ph.D.,^2,4^; David Erlinge, M.D., Ph.D.^5^; Troels Yndigegn, M.D.^5^; Joakim Alfredsson, M.D., Ph.D.^6^; Thomas Kellerth, M.D.^7^; Annica Ravn-Fischer, M.D., Ph.D.^8^ Sebastian Völz, M.D., Ph.D.^8^; Jörg Lauermann, M.D.^9^; Tomas Jernberg, M.D., Ph.D.^10^; Bertil Lindahl, M.D., Ph.D.^2,4^; and Sophie Langenskiöld, PhD,^2^ for the DETO2X-SWEDEHEART Investigators*

1. Department of Clinical Science and Education, Division of Cardiology, Karolinska Institutet, Södersjukhuset, Stockholm, Sweden.
2. Department of Medical Sciences, Uppsala University, Uppsala, Sweden
3. Department of Health Sciences, University of Borås, Borås, Sweden.
4. Uppsala Clinical Research Center, Uppsala University, Uppsala, Sweden.
5. Department of Clinical Sciences, Cardiology, Lund University, Lund, Sweden.
6. Department of Health, Medicine and Caring Sciences, Linköping University, and Department of Cardiology, Linköping University hospital, Linköping, Sweden.
7. Department of Cardiology, Faculty of Medicine and Health, Örebro University, Örebro
8. Department of Molecular and Clinical Medicine and Sahlgrenska University Hospital, Department of Cardiology, University of Gothenburg, Gothenburg, Sweden
9. Department of Cardiology, Jönköping, Region Jönköping County, and Department of Health, Medicine and Caring, Linköping University, Linköping, Sweden
10. Department of Clinical Sciences, Cardiology, Karolinska Institutet, Danderyd Hospital, Stockholm, Sweden.

**Table of contents**

| **STEMI** |  |
| --- | --- |
| Multivariate linear regression model of EQ-5D index and EQ-VAS score at 6-10 weeks (supplemental table I) | p 3 |
| EQ-5D dimensions at 6-10 weeks (supplemental table II) | p 5 |
| Multivariate linear regression model of EQ-5D index at 12-14 months (supplemental table III) | p 7 |
| EQ-5D dimensions at 12-14 months (supplemental table IV) | p 9 |
|  |  |
| **NSTEMI** |  |
| Multivariate linear regression model of EQ-5D index and EQ-VAS score at 6-10 weeks (supplemental table V) | p 11 |
| EQ-5D dimensions at 6-10 weeks (supplemental table VI) | p 13 |
|  |  |
| **Sensitivity analyses** |  |
| Multivariate linear regression model including hs-cTn T and SpO_2_ of EQ-5D index and EQ-VAS score at 6-10 weeks, all MI (supplemental table VII) | p 15 |
| Multivariate linear regression model including hs-cTn T and SpO_2_ of EQ-5D index and EQ-VAS score at 6-10 weeks, STEMI (supplemental table VIII) | p 17 |
| Multivariate linear regression model including hs-cTn T and SpO_2_ of EQ-5D index and EQ-VAS score at 6-10 weeks, NSTEMI (supplemental table IX) | p 19 |

**STEMI subgroup**

**Table I: Multivariate linear regression model of EQ-5D index and EQ-VAS score at 6-10 weeks for patients with STEMI**

|  | **EQ-5D index** | | | **EQ-VAS score** | | |
| --- | --- | --- | --- | --- | --- | --- |
| Coefficient | Estimates | 95% CI | p-value | Estimates | 95% CI | p-value |
| Intercept | 0.80 | 0.67 – 0.93 | <0.001 | 69.25 | 58.66 – 79.84 | <0.001 |
| **Intervention (Oxygen)** | -0.02 | -0.04 – 0.00 | 0.06 | **-1.33** | **-3.00 – 0.34** | **0.12** |
| Age (years) | 0.00 | 0.00 – 0.00 | <0.001 | 0.26 | 0.16 – 0.36 | <0.001 |
| Gender (female) | -0.07 | -0.09 – -0.04 | <0.001 | -2.60 | -4.63 – -0.57 | 0.01 |
| Body Mass Index (kg/m2) | -0.00 | -0.00 – 0.00 | 0.18 | -0.24 | -0.44 – -0.03 | 0.02 |
| Smoking (yes) | -0.05 | -0.07 – -0.03 | <0.001 | -2.87 | -4.71 – -1.03 | 0.002 |
| Hypertension (yes) | -0.02 | -0.05 – 0.01 | 0.31 | -2.89 | -5.39 – -0.38 | 0.02 |
| Diabetes (yes) | -0.02 | -0.06 – 0.01 | 0.13 | -1.35 | -3.94 – 1.25 | 0.31 |
| Previous MI (yes) | -0.05 | -0.12 – 0.01 | 0.11 | -3.94 | -9.25 – 1.37 | 0.15 |
| Previous PCI (yes) | 0.06 | -0.01 – 0.13 | 0.08 | 1.71 | -3.94 – 7.37 | 0.55 |
| Prior CABG (yes) | 0.02 | -0.06 – 0.11 | 0.61 | -1.34 | -8.35 – 5.66 | 0.71 |
| Aspirin (yes) | -0.03 | -0.07 – 0.01 | 0.16 | -0.91 | -4.35 – 2.53 | 0.61 |
| Beta-blockers (yes) | 0.00 | -0.03 – 0.03 | 0.96 | -0.03 | -2.75 – 2.69 | 0.98 |
| Statins (yes) | 0.00 | -0.03 – 0.03 | 0.97 | -0.41 | -3.23 – 2.42 | 0.78 |
| ACEI/ARB (yes) | 0.00 | -0.03 – 0.03 | 0.95 | 2.64 | 0.10 – 5.18 | 0.04 |
| CCB (yes) | -0.00 | -0.04 – 0.03 | 0.84 | 0.08 | -2.68 – 2.84 | 0.95 |
| Diuretic (yes) | -0.09 | -0.14 – -0.05 | <0.001 | -4.95 | -8.65 – -1.24 | 0.01 |
| Other antiplatelets (yes) | -0.01 | -0.10 – 0.08 | 0.91 | -3.29 | -10.86 – 4.28 | 0.39 |
| Ambulance service (yes) | 0.01 | -0.02 – 0.03 | 0.56 | -0.46 | -2.43 – 1.52 | 0.65 |
| Systolic BP (mmHg) | 0.00 | 0.00 – 0.00 | 0.03 | 0.03 | 0.00 – 0.07 | 0.03 |
| Heart rate (yeats/min) | -0.00 | -0.00 – 0.00 | 0.10 | -0.06 | -0.11 – -0.01 | 0.02 |
| Observations | 1719 | | | 1712 | | |
| R^2^ / R^2^ adjusted | 0.08 / 0.07 | | | 0.06 / 0.05 | | |

ACE denotes angiotensin converting enzyme; ARB, angiotensin receptor blocker; CABG, coronary artery bypass graft; CCB, calcium channel blockers; MI, myocardial infarction; PCI, percutaneous coronary intervention; STEMI, ST-segment elevation myocardial infarction.

**Table II: EQ-5D dimensions at 6-10 weeks in patients with STEMI**

|  | Visit 1 | | | | |
| --- | --- | --- | --- | --- | --- |
| Dimension | Oxygen  N=932 |  | Ambient air  N=1002 |  | p-value |
|  |  |  |  |  |  |
| MOBILITY n (%) |  |  |  |  | 0.09 |
| No problems | 731 (78.4) |  | 804 (80.2) |  |  |
| Some problems | 131 (14.1) |  | 123 (12.3) |  |  |
| Severe problems | 3 (0.3) |  | - |  |  |
| Missing | 67 (7.2) |  | 75 (7.5) |  |  |
| SELF-CARE n (%) |  |  |  |  | 0.52 |
| No problems | 850 (91.2) |  | 910 (90.8) |  |  |
| Some problems | 11 (1.2) |  | 15 (1.5) |  |  |
| Severe problems | 4 (0.4) |  | 2 (0.20) |  |  |
| Missing | 67 (7.2) |  | 75 (7.5) |  |  |
| USUAL ACTIVITIES n (%) |  |  |  |  | 0.55 |
| No problems | 728 (78.1) |  | 769 (79.4) |  |  |
| Some problems | 119 (12.8) |  | 116 (11.6) |  |  |
| Severe problems | 18 (1.9) |  | 15 (1.5) |  |  |
| Missing | 67 (7.2) |  | 75 (7.5) |  |  |
| PAIN/DISCOMFORT n (%) |  |  |  |  | **0.04** |
| No pain | 505 (54.2) |  | 596 (59.5) |  |  |
| Moderate pain | 328 (35.2) |  | 302 (30.1) |  |  |
| Severe Pain | 32 (3.4) |  | 29 (2.9) |  |  |
| Missing | 67 (7.2) |  | 75 (7.5) |  |  |
| ANXIETY/DEPRESSION n (%) |  |  |  |  | 0.24 |
| Not anxious or depressed | 513 (55.0) |  | 585 (58.4) |  |  |
| Moderately anxious or depressed | 318 (34.1) |  | 306 (30.5) |  |  |
| Severely anxious or depressed | 34 (3.7) |  | 36 (3.6) |  |  |
| Missing | 67 (7.2) |  | 75 (7.5) |  |  |

The EQ-5D is a generic instrument which measures patients’ health related quality of live five dimensions (mobility, self-care, usual activities, pain/discomfort, and anxiety/depression). The patients’ self-rate their current health state using a three-item ordinal response scale (no/moderate/severe problem).

**Table III: Multivariate linear regression model of EQ-5D index at 12-14 months in patients with STEMI**

|  | **EQ-5D index** | | |
| --- | --- | --- | --- |
| Coefficient | Estimates | 95% CI | p-value |
| Intercept | 0.87 | 0.73 – 1.00 | <0.001 |
| **Intervention (Oxygen)** | **-0.02** | **-0.04 – -0.00** | **0.03** |
| Age (years) | 0.00 | 0.00 – 0.00 | 0.02 |
| Gender (yemale) | -0.04 | -0.07 – -0.02 | 0.001 |
| Body Mass Index (Kg/m2) | -0.00 | -0.01 – -0.00 | <0.01 |
| Smoking (yes) | -0.04 | -0.06 – -0.02 | 0.001 |
| Hypertension (yes) | -0.02 | -0.05 – 0.01 | 0.23 |
| Diabetes (yes) | -0.08 | -0.11 – -0.04 | <0.001 |
| Previous MI (yes) | -0.06 | -0.12 – 0.01 | 0.09 |
| Previous PCI (yes) | 0.08 | 0.01 – 0.15 | 0.02 |
| Prior CABG (yes) | 0.06 | -0.02 – 0.14 | 0.17 |
| Aspirin (yes) | -0.08 | -0.12 – -0.04 | <0.001 |
| Beta-blockers (yes) | 0.02 | -0.02 – 0.05 | 0.28 |
| Statins (yes) | 0.01 | -0.03 – 0.04 | 0.62 |
| ACEI/ARB (yes) | 0.01 | -0.02 – 0.04 | 0.46 |
| CCB (yes) | 0.01 | -0.02 – 0.05 | 0.44 |
| Diuretic (yes) | -0.06 | -0.11 – -0.02 | <0.01 |
| Other antiplatelets (yes) | -0.00 | -0.10 – 0.10 | 0.97 |
| Ambulance service (yes) | -0.01 | -0.03 – 0.02 | 0.47 |
| Systolic BP (mmHg) | 0.00 | 0.00 – 0.00 | 0.001 |
| Heart rate (beats/min) | -0.00 | -0.00 – 0.00 | 0.21 |
| Observations | 1659 | | |
| R^2^ / R^2^ adjusted | 0.08 / 0.06 | | |

ACE denotes angiotensin converting enzyme; ARB, angiotensin receptor blocker; CABG, coronary artery bypass graft; CCB, calcium channel blockers; MI, myocardial infarction; PCI, percutaneous coronary intervention; STEMI, ST-segment elevation myocardial infarction.

**Table IV: EQ-5D dimensions at 12-14 months in patients with STEMI**

|  | Visit 2 | | | | |
| --- | --- | --- | --- | --- | --- |
| Dimension | Oxygen  N=932 |  | Ambient air  N=1002 |  | p-value |
|  |  |  |  |  |  |
| MOBILITY n (%) |  |  |  |  | 0.52 |
| No problems | 703 (75.4) |  | 736 (73.5) |  |  |
| Some problems | 128 (13.7) |  | 147 (14.7) |  |  |
| Severe problems | 4 (0.4) |  | 2 (0.20) |  |  |
| Missing | 97 (10.4) |  | 117 (11.7) |  |  |
| SELF-CARE n (%) |  |  |  |  | 0.96 |
| No problems | 814 (87.3) |  | 865 (86.3) |  |  |
| Some problems | 17 (1.8) |  | 16 (1.6) |  |  |
| Severe problems | 4 (0.4) |  | 4 (0.40) |  |  |
| Missing | 97 (10.4) |  | 117 (11.7) |  |  |
| USUAL ACTIVITIES n (%) |  |  |  |  | 0.78 |
| No problems | 727 (78.0) |  | 780 (77.8) |  |  |
| Some problems | 97 (10.4) |  | 93 (9.3) |  |  |
| Severe problems | 11 (1.2) |  | 12 (1.2) |  |  |
| Missing | 97 (10.4) |  | 117 (11.7) |  |  |
| PAIN/DISCOMFORT n (%) |  |  |  |  | 0.20 |
| No pain | 486 (52.1) |  | 547 (54.6) |  |  |
| Moderate pain | 309 (33.2) |  | 307 (30.6) |  |  |
| Severe Pain | 40 (4.3) |  | 31 (3.1) |  |  |
| Missing | 97 (10.4) |  | 117 (11.7) |  |  |
| ANXIETY/DEPRESSION n (%) |  |  |  |  | 0.12 |
| Not anxious or depressed | 540 (57.9) |  | 614 (61.3) |  |  |
| Moderately anxious or depressed | 270 (29.0) |  | 247 (24.7) |  |  |
| Severely anxious or depressed | 25 (2.7) |  | 24 (2.4) |  |  |
| Missing | 97 (10.4) |  | 117 (11.7) |  |  |

The EQ-5D is a generic instrument which measures patients’ health related quality of live five dimensions (mobility, self-care, usual activities, pain/discomfort, and anxiety/depression). The patients’ self-rate their current health state using a three-item ordinal response scale (no/moderate/severe problem).

**NSTEMI subgroup**

**Table V: Multivariate linear regression model of EQ-5D index and EQ-VAS score at 6-10 weeks in patients with NSTEMI**

|  | **EQ-5D index** | | | **EQ-VAS score** | | |
| --- | --- | --- | --- | --- | --- | --- |
| Coefficient | Estimates | 95%CI | p-value | Estimates | 95% CI | p-value |
| Intercept | 0.77 | 0.58 – 0.95 | <0.001 | 91.18 | 75.84 – 106.53 | <0.001 |
| **Intervention (Oxygen)** | **0.01** | **-0.02 – 0.04** | **0.64** | **2.19** | **-0.15 – 4.52** | **0.07** |
| Age (years) | 0.00 | 0.00 – 0.00 | <0.01 | 0.01 | -0.15 – 0.16 | 0.93 |
| Gender (female) | -0.04 | -0.07 – -0.00 | 0.04 | -1.67 | -4.63 – 1.28 | 0.27 |
| Body Mass Index (kg/m2) | -0.00 | -0.01 – -0.00 | 0.02 | -0.49 | -0.79 – -0.20 | 0.001 |
| Smoking (yes) | -0.05 | -0.08 – -0.01 | 0.01 | -7.30 | -10.14 – -4.46 | <0.001 |
| Hypertension (yes) | -0.03 | -0.07 – 0.01 | 0.15 | -2.37 | -5.72 – 0.98 | 0.17 |
| Diabetes (yes) | -0.04 | -0.08 – 0.00 | 0.07 | -5.11 | -8.47 – -1.75 | <0.01 |
| Previous MI (yes) | -0.06 | -0.13 – 0.01 | 0.07 | -4.98 | -10.49 – 0.52 | 0.08 |
| Previous PCI (yes) | 0.04 | -0.03 – 0.11 | 0.30 | 2.32 | -3.34 – 7.98 | 0.42 |
| Prior CABG (yes) | 0.08 | 0.01 – 0.16 | 0.02 | -0.94 | -6.76 – 4.89 | 0.75 |
| Aspirin (yes) | -0.03 | -0.07 – 0.02 | 0.26 | 3.39 | -0.27 – 7.05 | 0.07 |
| Beta-blockers (yes) | -0.03 | -0.07 – 0.02 | 0.23 | -3.27 | -6.70 – 0.15 | 0.06 |
| Statins (yes) | -0.03 | -0.07 – 0.01 | 0.19 | -1.33 | -4.78 – 2.11 | 0.45 |
| ACEI/ARB (yes) | 0.02 | -0.02 – 0.06 | 0.25 | 1.84 | -1.52 – 5.20 | 0.28 |
| CCB (yes) | 0.01 | -0.03 – 0.05 | 0.74 | 2.36 | -1.05 – 5.77 | 0.18 |
| Diuretic (Yes) | -0.03 | -0.08 – 0.02 | 0.20 | -3.55 | -7.58 – 0.47 | 0.08 |
| Other antiplatelets (yes) | -0.04 | -0.13 – 0.06 | 0.45 | -7.87 | -15.80 – 0.07 | 0.05 |
| Ambulance service (yes) | -0.01 | -0.04 – 0.02 | 0.57 | -2.76 | -5.14 – -0.37 | 0.02 |
| Systolic BP (mmHg) | 0.00 | 0.00 – 0.00 | 0.01 | 0.05 | 0.00 – 0.10 | 0.04 |
| Heart rate (beats/min) | -0.00 | -0.00 – 0.00 | 0.28 | -0.05 | -0.13 – 0.02 | 0.13 |
| Observations | 1006 | | | 905 | | |
| R^2^ / R^2^adjusted | 0.07 / 0.05 | | | 0.12 / 0.10 | | |

ACE denotes angiotensin converting enzyme; ARB, angiotensin receptor blocker; CABG, coronary artery bypass graft; CCB, calcium channel blockers; MI, myocardial infarction; PCI, percutaneous coronary intervention; STEMI, ST-segment elevation myocardial infarction.

**Table VI: EQ-5D dimensions at 6-10 weeks in patients with NSTEMI**

|  | Visit 1 | | | | |
| --- | --- | --- | --- | --- | --- |
| Dimension | Oxygen  n =586 |  | Ambient air  n=566 |  | p-value |
|  |  |  |  |  |  |
| MOBILITY n (%) |  |  |  |  | 0.57 |
| No problems | 451 (77.0) |  | 436 (77.0) |  |  |
| Some problems | 76 (13.0) |  | 82 (14.5) |  |  |
| Severe problems | 1 (0.2) |  | 2 (0.4) |  |  |
| Missing | 58 (9.9) |  | 46 (8.1) |  |  |
| SELF-CARE n (%) |  |  |  |  | 0.50 |
| No problems | 515 (87.9) |  | 508 (89.8) |  |  |
| Some problems | 11 (1.9) |  | 11 (1.9%) |  |  |
| Severe problems | 2 (0.3) |  | 1 (0.2) |  |  |
| Missing | 58 (9.9) |  | 46 (8.1) |  |  |
| USUAL ACTIVITIES n (%) |  |  |  |  | 0.22 |
| No problems | 443 (75.6) |  | 440 (77.7) |  |  |
| Some problems | 66 (11.3) |  | 70 (12.4) |  |  |
| Severe problems | 19 (3.2) |  | 10 (1.8) |  |  |
| Missing | 58 (9.9) |  | 46 (8.1) |  |  |
| PAIN/DISCOMFORT n (%) |  |  |  |  | 0.09 |
| No pain | 313 (53.4) |  | 310 (54.8) |  |  |
| Moderate pain | 194 (33.1) |  | 185 (32.7) |  |  |
| Severe Pain | 21 (3.6) |  | 25 (4.4) |  |  |
| Missing | 58 (9.9) |  | 46 (8.1) |  |  |
| ANXIETY/DEPRESSION n (%) |  |  |  |  | 0.34 |
| Not anxious or depressed | 338 (57.7) |  | 328(58.0) |  |  |
| Moderately anxious or depressed | 176 (30.0) |  | 171 (30.2) |  |  |
| Severely anxious or depressed | 14 (2.4) |  | 21 (3.2) |  |  |
| Missing | 58 (9.9) |  | 46 (8.1) |  |  |

The EQ-5D is a generic instrument which measures patients’ health related quality of live five dimensions (mobility, self-care, usual activities, pain/discomfort, and anxiety/depression). The patients’ self-rate their current health state using a three-item ordinal response scale (no/moderate/severe problem).

**SENSITIVITY ANALYSES**

**Table VII: Multivariate linear regression model including hs-cTn T and SpO_2_ of EQ-5D index and EQ-VAS score at 6-10 weeks in MI patients**

|  | **EQ-5D index** | | | **EQ-VAS score** | | |
| --- | --- | --- | --- | --- | --- | --- |
| Coefficient | Estimates | 95% CI | p-value | Estimates | 95% CI | p-value |
| Intercept | 0.48 | 0.02 – 0.95 | 0.04 | 25.02 | 12.29 – 62.33 | 0.19 |
| **Intervention (Oxygen)** | **-0.01** | **-0.03 – 0.01** | **0.37** | **-0.38** | **-1.90 – 1.13** | **0.62** |
| Age (years) | 0.00 | 0.00 – 0.00 | <0.001 | 0.27 | 0.18 – 0.37 | <0.001 |
| Gender (female) | -0.06 | -0.08 – -0.04 | <0.001 | -2.46 | -4.32 – -0.59 | 0.01 |
| Body Mass Index (Kg/m2) | -0.00 | -0.00 – -0.00 | 0.03 | -0.27 | -0.45 – -0.08 | 0.01 |
| Smoking (yes) | -0.04 | -0.07 – -0.02 | <0.001 | -3.19 | -4.91 – -1.47 | <0.001 |
| Hypertension (yes) | -0.02 | -0.05 – 0.01 | 0.23 | -1.85 | -4.10 – 0.40 | 0.11 |
| Diabetes (yes) | -0.03 | -0.06 – -0.00 | 0.04 | -2.29 | -4.59 – 0.01 | 0.05 |
| Previous MI (yes) | -0.04 | -0.09 – 0.01 | 0.16 | -3.81 | -8.04 – 0.43 | 0.08 |
| Previous PCI (yes) | 0.03 | -0.03 – 0.08 | 0.30 | 1.62 | -2.76 – 6.00 | 0.47 |
| Prior CABG (yes) | 0.05 | -0.01 – 0.11 | 0.12 | 0.24 | -4.55 – 5.03 | 0.92 |
| Aspirin (yes) | -0.03 | -0.06 – 0.01 | 0.12 | 1.72 | -1.07 – 4.50 | 0.23 |
| Beta blockers (yes) | -0.01 | -0.03 – 0.02 | 0.73 | -1.00 | -3.40 – 1.40 | 0.41 |
| Statins (yes) | -0.03 | -0.06 – 0.01 | 0.10 | -2.94 | -5.45 – -0.43 | 0.02 |
| ACEI/ARB (yes) | 0.01 | -0.02 – 0.04 | 0.43 | 2.26 | -0.01 – 4.52 | 0.05 |
| CCB (yes) | 0.00 | -0.03 – 0.03 | 0.81 | 0.74 | -1.68 – 3.15 | 0.55 |
| Diuretic (yes) | -0.06 | -0.10 – -0.02 | 0.003 | -4.77 | -7.81 – -1.74 | 0.002 |
| Other antiplatelets (yes) | -0.04 | -0.11 – 0.04 | 0.34 | -4.63 | -10.79 – 1.52 | 0.14 |
| Ambulance service (yes) | -0.00 | -0.02 – 0.02 | 0.85 | -1.25 | -2.95 – 0.45 | 0.15 |
| Systolic BP (mmHg) | 0.00 | 0.00 – 0.00 | 0.004 | 0.04 | 0.01 – 0.07 | 0.004 |
| Heart rate (beats/min) | -0.00 | -0.00 – 0.00 | 0.08 | -0.04 | -0.08 – 0.01 | 0.09 |
| MI subtype (NSTEMI) | -0.01 | -0.03 – 0.01 | 0.39 | 0.05 | -1.71 – 1.81 | 0.96 |
| Oxygen saturation (%) | 0.00 | -0.00 – 0.01 | 0.16 | 0.43 | 0.07 – 0.78 | 0.02 |
| Troponin level (ng/ml) | 0.00 | -0.00 – 0.00 | 0.68 | -0.00 | -0.00 – 0.00 | 0.72 |
| Observations | 2180 | | | 2177 | | |
| R^2^ / R^2^ adjusted | 0.07 / 0.06 | | | 0.07 / 0.06 | | |

ACE denotes angiotensin converting enzyme; ARB, angiotensin receptor blocker; CABG, coronary artery bypass graft; CCB, calcium channel blockers; MI, myocardial infarction; PCI, percutaneous coronary intervention; STEMI, ST-segment elevation myocardial infarction.

**Table VIII: Multivariate linear regression model including hs-cTn T and SpO_2_ of EQ-5D index and EQ-VAS score at 6-10 weeks in patients with STEMI**

|  | **EQ-5D index** | | | **EQ-VAS score** | | |
| --- | --- | --- | --- | --- | --- | --- |
| Coefficient | Estimates | 95%CI | p-value | Estimates | 95%CI | p-value |
| Intercept | 0.08 | -0.43 – 0.60 | 0.75 | 5.61 | -37.79 – 49.01 | 0.80 |
| **Intervention (Oxygen)** | **-0.01** | **-0.04 – 0.01** | **0.23** | **-0.76** | **-2.64 – 1.11** | **0.43** |
| Age (years) | 0.00 | 0.00 – 0.00 | <0.001 | 0.34 | 0.22 – 0.45 | <0.001 |
| Gender (female) | -0.07 | -0.10 – -0.04 | <0.001 | -2.96 | -5.23 – -0.68 | 0.01 |
| Body Mass Index (kg/m2) | -0.00 | -0.00 – 0.00 | 0.34 | -0.22 | -0.45 – 0.01 | 0.06 |
| Smoking (yes) | -0.05 | -0.07 – -0.02 | <0.001 | -2.24 | -4.30 – -0.17 | 0.03 |
| Hypertension (yes) | -0.02 | -0.05 – 0.02 | 0.36 | -3.62 | -6.44 – -0.81 | 0.01 |
| Diabetes (yes) | -0.03 | -0.06 – 0.01 | 0.15 | -1.47 | -4.40 – 1.46 | 0.33 |
| Previous MI (yes) | -0.04 | -0.11 – 0.03 | 0.23 | -2.83 | -8.60 – 2.94 | 0.34 |
| Previous PCI (yes) | 0.05 | -0.03 – 0.12 | 0.21 | 2.71 | -3.44 – 8.86 | 0.39 |
| Prior CABG (yes) | 0.02 | -0.07 – 0.12 | 0.63 | -0.63 | -8.38 – 7.13 | 0.87 |
| Aspirin (yes) | -0.04 | -0.09 – 0.01 | 0.10 | -1.70 | -5.68 – 2.29 | 0.40 |
| Beta blockers (yes) | 0.01 | -0.03 – 0.04 | 0.74 | -0.36 | -3.48 – 2.76 | 0.82 |
| Statins (yes) | -0.01 | -0.05 – 0.03 | 0.77 | -1.36 | -4.65 – 1.93 | 0.42 |
| ACEI/ARB (yes) | 0.00 | -0.03 – 0.04 | 0.91 | 3.22 | 0.33 – 6.11 | 0.03 |
| CCB (yes) | 0.00 | -0.03 – 0.04 | 0.86 | 1.19 | -1.98 – 4.36 | 0.46 |
| Diuretic (yes) | -0.09 | -0.15 – -0.04 | <0.001 | -5.55 | -9.83 – -1.26 | 0.01 |
| Other antiplatelets (yes) | -0.01 | -0.11 – 0.09 | 0.83 | -1.72 | -10.21 – 6.78 | 0.69 |
| Ambulance service (yes) | 0.00 | -0.02 – 0.03 | 0.72 | -0.44 | -2.70 – 1.82 | 0.70 |
| Systolic BP (mmHg) | 0.00 | 0.00 – 0.00 | 0.03 | 0.04 | 0.00 – 0.07 | 0.03 |
| Heart rate (beats/min) | -0.00 | -0.00 – 0.00 | 0.10 | -0.05 | -0.10 – 0.01 | 0.09 |
| Oxygen saturation (%) | 0.01 | 0.00 – 0.01 | <0.01 | 0.59 | 0.18 – 1.00 | <0.01 |
| Troponin level (ng/ml) | 0.00 | -0.00 – 0.00 | 0.59 | -0.00 | -0.00 – 0.00 | 0.55 |
| Observations | 1421 | | | 1418 | | |
| R^2^ / R^2^adjusted | 0.08 / 0.07 | | | 0.07 / 0.06 | | |

ACE denotes angiotensin converting enzyme; ARB, angiotensin receptor blocker; CABG, coronary artery bypass graft; CCB, calcium channel blockers; MI, myocardial infarction; PCI, percutaneous coronary intervention; STEMI, ST-segment elevation myocardial infarction.

**Table IX: Multivariate linear regression model including hs-cTn T and SpO_2_ of EQ-5D index and EQ-VAS score at 6-10 weeks in patients with NSTEMI**

|  | **EQ-5D index** | | | **EQ-VAS score** | | |
| --- | --- | --- | --- | --- | --- | --- |
| Coefficient | Estimates | 95% CI | p-value | Estimates | 95% CI | p-value |
| Intercept | 1.60 | 0.61 – 2.58 | 0.001 | 106.19 | 27.95 – 184.43 | 0.01 |
| **Intervention (Oxygen)** | **0.00** | **-0.03 – 0.04** | **0.89** | **2.62** | **-0.14 – 5.37** | **0.06** |
| Age (years) | 0.00 | 0.00 – 0.01 | 0.01 | -0.01 | -0.20 – 0.18 | 0.93 |
| Gender (female) | -0.04 | -0.08 – 0.01 | 0.10 | -1.07 | -4.62 – 2.49 | 0.56 |
| Body Mass Index (kg/m2) | -0.00 | -0.01 – -0.00 | 0.04 | -0.60 | -0.96 – -0.24 | 0.001 |
| Smoking (yes) | -0.04 | -0.08 – 0.00 | 0.08 | -7.24 | -10.65 – -3.83 | <0.001 |
| Hypertension (yes) | -0.02 | -0.07 – 0.03 | 0.42 | -0.36 | -4.51 – 3.78 | 0.86 |
| Diabetes (yes) | -0.03 | -0.08 – 0.02 | 0.28 | -3.60 | -7.58 – 0.38 | 0.08 |
| Previous MI (yes) | -0.04 | -0.13 – 0.04 | 0.32 | -3.19 | -10.09 – 3.71 | 0.36 |
| Previous PCI (yes) | 0.02 | -0.06 – 0.11 | 0.60 | -1.65 | -8.41 – 5.12 | 0.63 |
| Prior CABG (yes) | 0.08 | -0.00 – 0.16 | 0.07 | -1.74 | -8.47 – 4.99 | 0.61 |
| Aspirin (yes) | -0.02 | -0.08 – 0.03 | 0.39 | 4.83 | 0.53 – 9.13 | 0.03 |
| Beta-blockers (yes) | -0.02 | -0.07 – 0.03 | 0.49 | -3.98 | -8.05 – 0.08 | 0.06 |
| Statins (yes) | -0.05 | -0.11 – -0.00 | 0.04 | -1.34 | -5.47 – 2.80 | 0.53 |
| ACEI/ARB (yes) | 0.02 | -0.03 – 0.07 | 0.44 | -0.54 | -4.60 – 3.53 | 0.80 |
| CCB (yes) | 0.01 | -0.04 – 0.06 | 0.72 | 2.08 | -1.96 – 6.13 | 0.31 |
| Diuretic (yes) | -0.03 | -0.09 – 0.03 | 0.33 | -2.49 | -7.21 – 2.23 | 0.30 |
| Other antiplatelets (yes) | -0.05 | -0.18 – 0.07 | 0.38 | -4.51 | -14.73 – 5.70 | 0.37 |
| Ambulance service (yes) | -0.01 | -0.05 – 0.02 | 0.40 | -3.19 | -5.99 – -0.40 | 0.03 |
| Systolic BP (mmHg) | 0.00 | -0.00 – 0.00 | 0.07 | 0.07 | 0.01 – 0.13 | 0.01 |
| Heart rate (beats/min) | -0.00 | -0.00 – 0.00 | 0.44 | -0.08 | -0.17 – 0.00 | 0.06 |
| Oxygen saturation (%) | -0.01 | -0.02 – 0.00 | 0.08 | -0.14 | -0.88 – 0.60 | 0.71 |
| Troponin level (ng/ml) | -0.00 | -0.00 – 0.00 | 0.69 | 0.00 | -0.00 – 0.00 | 0.40 |
| Observations | 759 | | | 680 | | |
| R^2^ / R^2^adjusted | 0.07 / 0.04 | | | 0.12 / 0.09 | | |

ACE denotes angiotensin converting enzyme; ARB, angiotensin receptor blocker; CABG, coronary artery bypass graft; CCB, calcium channel blockers; MI, myocardial infarction; PCI, percutaneous coronary intervention; STEMI, ST-segment elevation myocardial infarction.
